# Autophagy-related circRNA evaluation reveals hsa_circ_0001747 as a potential favorable prognostic factor for biochemical recurrence in patients with prostate cancer

**DOI:** 10.1038/s41419-021-04015-w

**Published:** 2021-07-22

**Authors:** Chuanfan Zhong, Kaihui Wu, Shuo Wang, Zining Long, Taowei Yang, Weibo Zhong, Xiao Tan, Zixian Wang, Chuanyin Li, Jianming Lu, Xiangming Mao

**Affiliations:** 1 Department of Urology, Zhujiang Hospital, Southern Medical University, Guangzhou, China; 2 Irvine Valley College, Irvine, CA USA

**Keywords:** Tumour biomarkers, Macroautophagy

## Abstract

Prostate cancer (PCa) is a common high-incidence malignancy in men, some of whom develop biochemical recurrence (BCR) in the advanced stage. However, there are currently no accurate prognostic indicators of BCR in PCa. The aim of our study was to identify an autophagy-related circular RNA prognostic factor of BCR for patients with PCa. In this study, immunochemistry revealed that the classic autophagy marker *MAP1LC3B* was positively correlated with Gleason score. Least absolute shrinkage and selector operator regression were conducted to develop a novel prognostic model with tenfold cross-validation and an L1 penalty. Five autophagy-related circRNA signatures were included in the prognostic model. Patients with PCa were ultimately divided into high- and low-risk groups, based on the median risk score. Patients with PCa, who had a high risk score, were more likely to develop BCR in a shorter period of time. Univariate and multivariate Cox regression analyses demonstrated that the risk score was an independent variable for predicting BCR in PCa. In addition, a prognostic nomogram integrated with the risk score and numerous clinicopathological parameters was developed to accurately predict 3- and 5-year BCR of patients with PCa. Finally, the hsa_circ_0001747 signature was selected for further experimental verification in vitro and in vivo, which showed that downregulated hsa_circ_0001747 might facilitate PCa via augmenting autophagy. Our findings indicate that the autophagy-related circRNA signature hsa_circ_0001747 may serve as a promising indicator for BCR prediction in patients with PCa.

## Introduction

Prostate cancer (PCa) is one of the common solid tumors in men and it is ranked as first in incidence and second in mortality among all male tumors in North America [1]. Various assessment indexes, including the Gleason score (GS), pathological TNM stage, marginal positivity, and pre-operation prostate-specific antigen (PSA) level, are commonly used in PCa diagnosis and prognostic prediction; however, over one-third of patients eventually experience biochemical recurrence (BCR) after surgery [2, 3]. Therefore, more effective and precise evaluation approaches are urgently needed to predict BCR.

Autophagy, traditionally referred to as macroautophagy, is a conserved ubiquitous process and material recycling system that disposes damaged organelles, misfolded proteins, and intracellular constituents in lysosome-dependent degradation. However, autophagy has a double role in cell survival regulation [4, 5]. On one hand, autophagy sustains cell homeostasis via recycling substances or energy within the cell and survival through unfavorable environments, such as hypoxia, starvation, and drug stress [6, 7]. On the other hand, excessive autophagy activation will overconsume cell materials, thereby inducing cell death [8]. Various studies have shown that autophagy is enhanced in numerous types of tumors, such as breast cancer, small cell lung cancer, and ovarian cancer [9–11]. However, autophagy can be cytotoxic to cancerous cells, driving them to programmed cell death [12, 13]. Recently, a phase II clinical trial was conducted, using pantoprazole (an autophagy inhibitor targeting the proton pump) combined with docetaxel in the treatment of metastatic castration-resistant PCa; however, most cases were observed with limited clinical response [14]. Autophagy is a complex biological process that is closely related to the occurrence and progression of cancers, including PCa. Simple treatment with autophagy inhibitors often fails to meet clinical expectations. Thus, identifying a group of novel molecules that can directly or indirectly regulate autophagy is urgently needed in cancer diagnosis and therapy.

Genome sequencing studies have shown that <5% of human transcripts have encoding potential, over 90% of which are non-coding RNA (ncRNA) [15]. Among all types of ncRNAs, circular RNA (circRNA) is a covalently closed ncRNA that is currently identified to have temporal and spatial expression specificity, as well as tissue specificity [16]. Depending on its sub-position, circRNA is inclined to function as a sponge of microRNAs or a scaffold of proteins in the cytoplasm, while directly regulating chromatin remodeling in the nucleus [17, 18]. It is widely reported that circRNA plays an essential role in the carcinogenesis, aggressiveness, and drug resistance of various tumors. For example, it is reported that circRNA MAT2B disrupts the degradation of *PKM2* via miR-338-3p sponging, thereby enhancing glycolysis to promote hepatocellular carcinoma progression under hypoxic stress [19]. Jie et al. [20] revealed that circMRPS35 not only recruits histone acetyltransferase *KAT7* to the promoters of *FOXO1/3a* but also binds directly to the *FOXO1/3a* protein, which inhibits the metastasis of gastric cancer. Moreover, circRNA is closely related to autophagy in cancers, which has been widely studied recently [21]. For example, through competing endogenous RNA (ceRNA) function, circPARD3 drives chemoresistance in laryngeal squamous cell carcinoma by activating *PRKCI-AKT-mTOR* pathway-induced autophagy [22]. However, whether circRNA acts as a stimulator or inhibitor of autophagy in PCa remains unclear.

Rapid advances in transcriptome sequencing technology and bioinformatics have provided new insights for identifying potential biomarkers that can predict the prognosis of patients with cancer [23, 24]. Thus, we hypothesized that autophagy-related circRNAs can be valuable prognostic biomarkers for patients with PCa. In our study, we confirmed that the classic autophagy marker *MAP1LC3B* was highly expressed in PCa tissues and correlated with a high GS in tissue microarray (TMA). We then screened differentially expressed autophagy-related circRNAs in Pearson’s correlation analysis of 144 patients with PCa and constructed a prognostic model based on five autophagy-related circRNAs (hsa_circ_0000280, hsa_circ_0000437, hsa_circ_0001085, hsa_circ_0002100, and hsa_circ_0001747) via least absolute shrinkage and selector operator (LASSO) regression. Finally, using univariate and multivariate Cox regression analysis and Kaplan–Meier (KM) plots, as well as in vitro and in vivo experiments, we identified hsa_circ_0001747 as having a vital role in the regulation of autophagy in PCa.

## Results

### High expression level of *MAP1LC3B* protein is associated with aggressiveness of human PCa

**Fig. 1:**
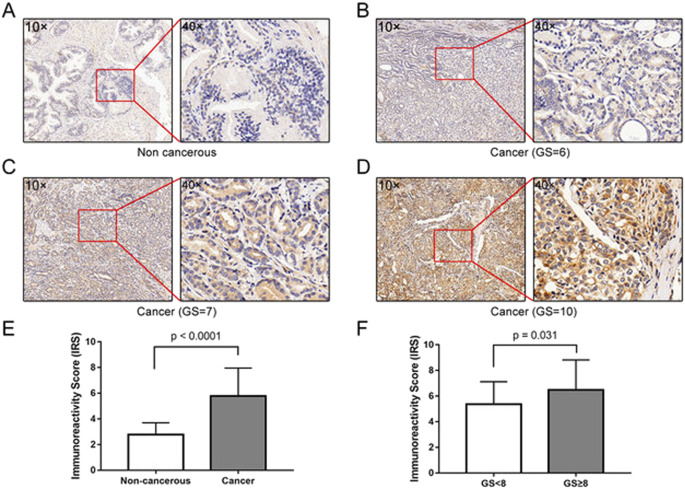
Immunostaining of *MAP1LC3B* in PCa and normal prostate tissues. **A** Low expression of *MAP1LC3B* detected in normal prostate tissues. **B**–**D** High expression of *MAP1LC3B* detected in prostate cancer tissues. **E**, **F** Statistical analysis revealed *MAP1LC3B* was lowly expressed in non-cancerous prostate tissues, whereas highly expressed in PCa tissues, and correlated positively with Gleason score.

The expression and subcellular localization of *MAP1LC3B* protein in 80 cases of PCa tissues and 14 cases of non-cancerous prostate tissues were detected using immunohistochemistry (IHC). The positive immunostaining areas of *MAP1LC3B* were mainly in the cytoplasm and cell membranes of PCa and normal prostate cells (Fig. 1A–D). The level of *MAP1LC3B* protein in PCa tissues was higher than that in non-cancerous prostate tissues (*P* < 0.001, 5.86 ± 2.25 vs. 2.78 ± 0.92) (Fig. 1E). In 80 patients with PCa, *MAP1LC3B* protein level was positively correlated with GS (*P* = 0.031, immunoreactive score, immunoreactivity score (IRS) of GS < 8 was 5.36 ± 1.75, and IRS of GS ≥ 8 was 6.48 ± 2.34), but not with the pathological stage, and metastasis (Fig. 1F and Table 1). This suggested that autophagy might play a vital role in aggressive PCa progression.

**Table 1:** Correlation between *MAP1LC3B* protein expression level and patients clinicopathological characteristics.

	*N*	Mean ± SD	*P*-value
Type of tissue			
Non-cancerous	14	2.78 ± 0.92	<0.001
Cancer	80	5.86 ± 2.25	
Gleason score			
<8	29	5.36 ± 1.75	0.031
≥8	46	6.48 ± 2.34	
Pathological stage			
≤T2c	51	5.98 ± 2.13	0.556
T3a–T4	29	5.65 ± 2.43	
Metastasis			
Yes	8	5.01 ± 1.73	0.260
No	72	5.95 ± 2.29	

### Identification of autophagy-related circRNAs in PCa

**Fig. 2:**
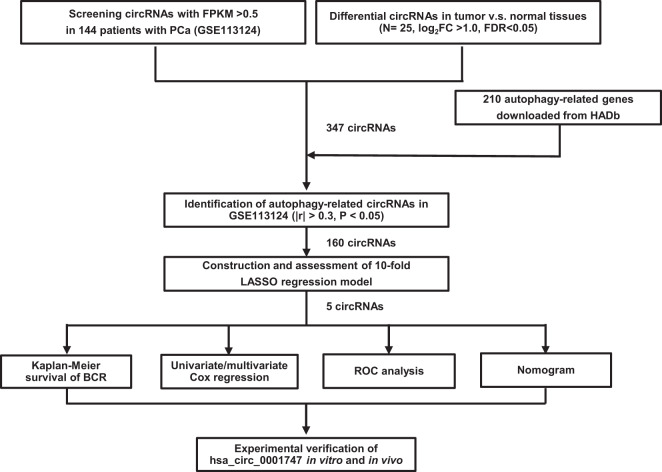
Flow chart of autophagy-related circRNA signature screening and validation.

Figure 2 shows the flow chart for identifying autophagy-related circRNAs in this study. As autophagy has a multifaceted regulating role in cancers, we followed the rationale to identify novel autophagy-related modulators that were expected to be applied to clinical diagnosis, survival prediction, and even therapeutic alternatives. In comparative analysis of circRNA expression between 25 pairs of PCa tissues and adjacent normal tissues, we identified 877 significantly differentially expressed circRNAs (the absolute value of logFC > 1, false discovery rate (FDR) < 0.05), which included 34 upregulated and 843 downregulated circRNAs (Supplemental Fig. S1A). Then, we compared 1199 circRNAs (fragments per kilobase of per million (FPKM) > 0.5 from GSE113124) with 877 differentially expressed circRNAs, resulting in 347 common circRNAs (Supplemental Table S1). Finally, using Pearson’s correlation analysis between these 347 circRNAs and 210 autophagy-related genes from The Human Autophagy Database (HADb) website, we obtained 160 autophagy-related circRNAs (Supplemental Table S1).

### Construction and evaluation of autophagy-associated circRNA signature in PCa

**Fig. 3:**
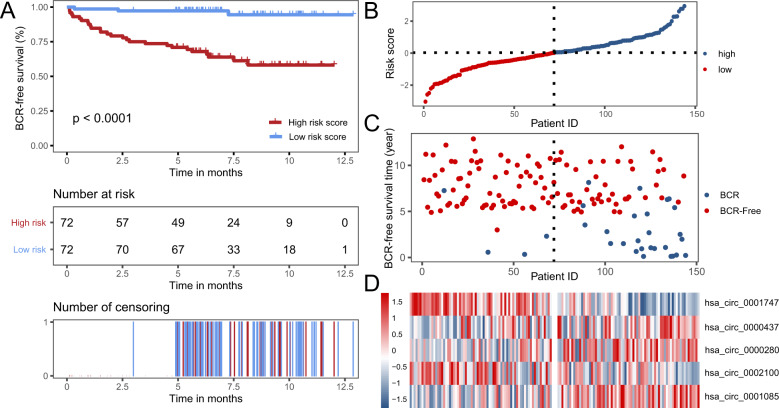
Construction and validation of autophagy-related circRNA signature in PCa patients. **A** KM survival curve analysis shows that BCR-free survival time of patients with high-risk scores is significantly shorter than those with low-risk scores. **B** Distribution of risk scores of high- and low-risk patients with PCa. **C** Scatterplot shows the correlation between survival time and risk score of patients with PCa. **D** Heatmap shows that high-risk patients displayed higher level of risk factors (hsa_circ_0000437, hsa_circ_0000280, and circ_5017), whereas low-risk patients displayed higher level of protective factors (hsa_circ_0001747 and hsa_circ_0002100).

We applied tenfold cross-validation LASSO regression to construct the prognostic signature, which is based on the clinical data and expression levels of 160 autophagy-related circRNAs in PCa samples (Supplemental Fig. S2A, B). According to the independent regression coefficients of five autophagy-related circRNAs, we calculated the risk score of each sample in GSE113124. The formula is as follows: Risk score = 0.060 × hsa_circ_0000280 + 0.004 × hsa_circ_0000437 + 0.032 × hsa_circ_0001085 − 0.006 × hsa_circ_0002100 − 0.075 × hsa_circ_0001747. The essential information of these five circRNAs is shown in Table 2. Then, patients were divided into a high-risk or low-risk group with the median risk score of −0.144. The KM survival curve demonstrated that patients in the low-risk group had significantly better BCR-free survival than those in the high-risk group (*P*-value < 0.0001) (Fig. 3A). The risk curve of risk score profiles and scatterplot of BCR status described that high-risk patients had shorter BCR-free times than low-risk patients (Fig. 3B, C), which was in accord with the KM survival curve. A heatmap of the five autophagy-related circRNA signatures showed that hsa_circ_0001747 and hsa_circ_0002100 were highly expressed in the low-risk group, whereas hsa_circ_0000280, hsa_circ_0000437, and hsa_circ_0001085 were significantly expressed in the high-risk group (Fig. 3D).

**Table 2:** Recent studies of five autophagy-related circRNAs and their corresponding host genes.

CircRNA	Gene symbol	Description	Associated diseases of host gene	Associated diseases of circRNA
hsa_circ_0000280	*HPS5*	Hermansky–Pudlak Syndrome 5	Hermansky–Pudlak type 5 syndrome [43]	
hsa_circ_0000437	*CORO1C*	Coronin, Actin-binding protein, 1C	Gastric cancer [44] Hepatocellular carcinoma [45] Lung cancer [46] Breast Cancer [47]	Endometrial cancer [48]
has_circ_0001085	*GLS*	Glutaminase	Prostate cancer [49] Cholangiocarcinoma [50] Non-small cell lung cancer [51] Colorectal cancer [52] Breast cancer [53]	--
hsa_circ_0002100	*ARHGEF12*	Rho guanine nucleotide exchange factor 12	Acute myeloid leukemia [54] Glaucoma [55] Acute lymphoblastic leukemia [56]	--
hsa_circ_0001747	*MKLN1*	Muskelin 1	Bipolar disorder [57]	Tuberculosis [58]

### Autophagy-related circRNA signature is an independent prognostic indicator

**Fig. 4:**
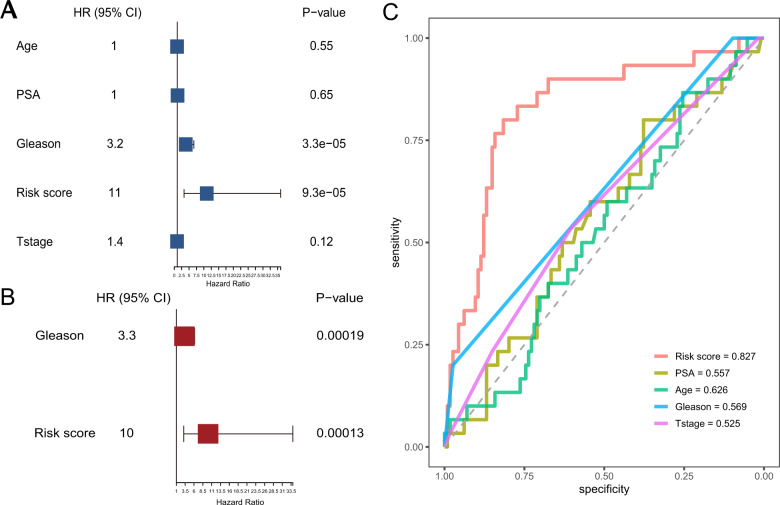
Estimation of the prognostic accuracy of the autophagy-related circRNA signature and other clinicopathological factors in PCa patients. **A** Univariate Cox regression analysis shows the correlation between BCR-free survival and various clinicopathological parameters such as age, PSA, T-stage, GS, and the autophagy-related circRNA signature risk score. **B** Multivariate Cox regression analysis shows the correlation between BCR-free survival with GS and the autophagy-related circRNA signature risk score. **C** ROC analysis shows the prognostic accuracy of clinicopathological parameters such as age, PSA, T-stage, GS, and the autophagy-related circRNA signature risk score.

Next, we applied univariate and multivariate Cox regression analysis to evaluate whether the autophagy-related circRNA signature was an independent prognostic indicator in patients with PCa. Univariate Cox regression analysis showed that GS (*P*-value = 0.000033) and autophagy-related prognostic risk score (*P*-value = 0.000093) were significantly associated with BCR-free survival (Fig. 4A and Supplemental Table S3). Multivariate Cox regression analysis showed that both GS (*P*-value = 0.00019) and autophagy-related prognostic risk score (*P*-value = 0.00013) were significantly associated with BCR-free survival (Fig. 4B and Supplemental Table S3). We also conducted receiver operating characteristic (ROC) curve analysis to estimate the prognostic reliability of the risk score model. As depicted in Fig. 4C, the area under the ROC curve (AUC) value for the autophagy-related circRNA signature was 0.827, which was higher than the AUC values for PSA (AUC = 0.557), age (AUC = 0.626), GS (AUC = 0.569), and T-stage (AUC = 0.525). These data demonstrated that the autophagy-related circRNA prognostic signature is an independent prognostic indicator for patients with PCa.

### Evaluation of prognostic prediction nomogram including autophagy-related circRNA prognostic signature risk score

**Fig. 5:**
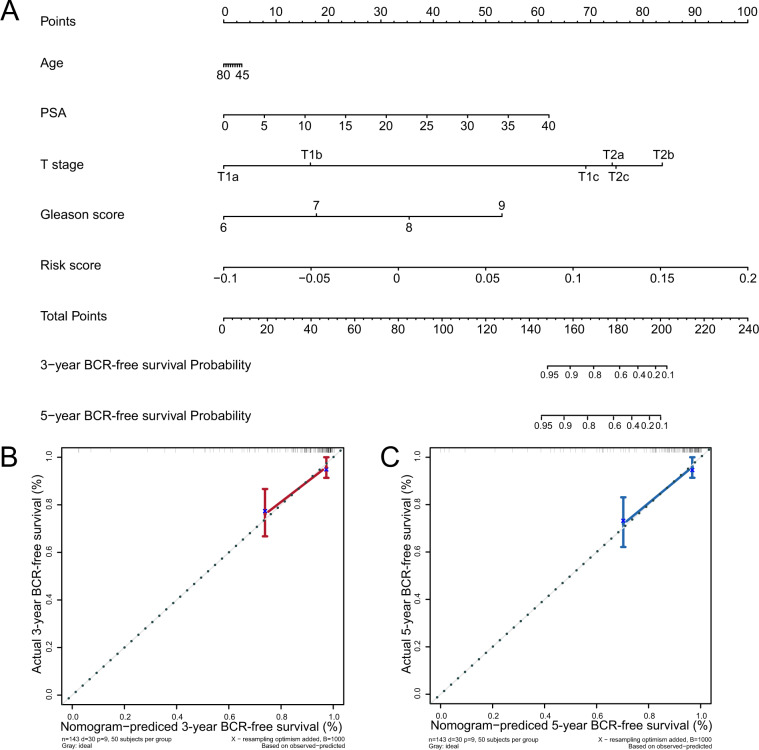
Construction of a nomogram for BCR-free survival prediction. **A** The nomogram containing the risk score and clinicopathological features. **B**, **C** Calibration plot showing that nomogram-predicted 3- and 5-year BCR-free survival probabilities were in accordance with the actual observed proportions.

Nomograms are widely used by clinicians in predicting survival for patients by calculating the set points based on each nomogram score. In this study, we built a nomogram to predict 3- and 5-year BCR-free survival for any PCa patient based on the scores of predictive variables including PSA, age, T-stage, GS, and five autophagy-related circRNAs risk score (Fig. 5A). Based on the value each variable of a patient, we find the corresponding points by comparing the “Points” axis with the axis for that variable. Total points of this patient can be calculated by summing up these points of predictive variables. Finally, the probabilities of 3- or 5-year BCR-free survival for the patient can be identified based on the total points by comparing the “Total Points” axis with axes for the probabilities of 3- or 5-year BCR-free survival, respectively. We also constructed a calibration curve analysis and the results demonstrated that the 3- and 5-year BCR-free survival estimated by the nomogram was in accordance with the actual condition (Fig. 5B, C). All findings elucidated that the five autophagy-related circRNA signatures for patients with PCa were highly accurate and reliable.

### Individual analysis of the five autophagy-related circRNAs and screening of hsa_circ_0001747 for experimental validation

**Fig. 6:**
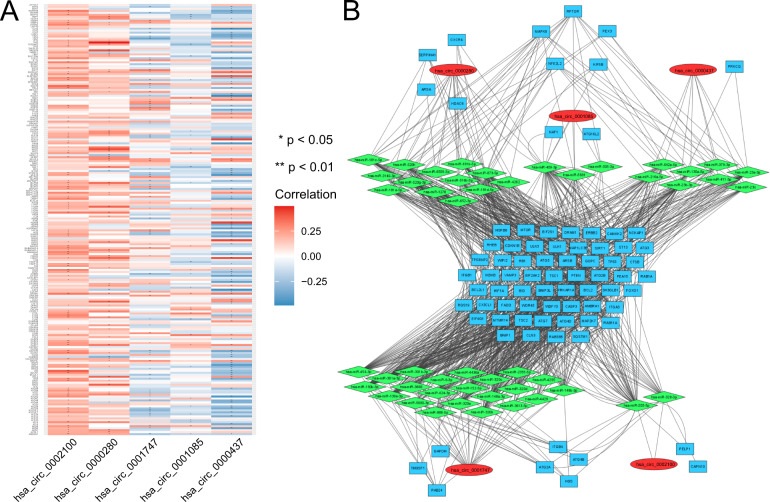
Correlation and network of five autophagy-related circRNAs, downstream miRNAs, and autophagy-related genes. **A** Heatmap of correlation between five circRNAs and 210 autophagy-related genes. **B** CeRNA network analysis of the five circRNAs, downstream miRNAs and miRNA-targeted autophagy-related genes.

As shown in Supplemental Fig. S2A–E, all autophagy-related circRNAs were generally downregulated in PCa tissues. We used KM plots to evaluate the relationship between individual expression of autophagy-related circRNAs and BCR-free survival in patients with PCa. The results showed that high expression of hsa_circ_0001747 predicts longer BCR-free time (Supplemental Fig. S3A), whereas low expression of hsa_circ_0000437 and hsa_circ_0000280 predicts shorter BCR-free time (Supplemental Fig. S3B, C). However, the expressions of hsa_circ_0002100 and hsa_circ_0001085 were not significantly associated with BCR-free survival in patients with PCa, although both displayed a prognostic trend in KM plots (Supplemental Fig. S3D, F). Then, we conducted univariate and multivariate Cox analysis of the five autophagy-related circRNAs based on BCR status, using the “forestplot” package. Notably, has_circ_0001747 with hazard ratio (HR) < 1.0 and hsa_circ_0000280 with HR > 1.0 were significantly independent prognostic factors for BCR status (Supplemental Fig. S4A, B). In addition, we applied a heatmap of the correlation between circRNAs and autophagy-related genes based on their correlation coefficients. As shown in Fig. 6A, hsa_circ_0001747, hsa_circ_0001085, and hsa_circ_0000437 were negatively correlated with autophagy-related genes, whereas hsa_circ_0002100 and hsa_0000280 were positively correlated with these. The miRNA often binds to the 3′-untranslated region of targeted messenger RNA (mRNA) and inhibits its translation, whereas circRNA generally functions as a miRNA sponge due to its unique circular structure, leading to removal of miRNA inhibition. Therefore, a ceRNA network analysis was leveraged to visualize the relationship of these five circRNAs, their predicted downstream miRNAs, and autophagy-related genes. As depicted in Fig. 6B, the five autophagy-related circRNAs had a set of independent miRNAs but modulated a common part of downstream autophagy-related genes (middle of Fig. 6B). In addition, they were also divided into two subgroups including subgroup 1 (hsa_circ_0002100 and hsa_circ_0001747) and subgroup 2 (hsa_circ_0001085, hsa_circ_0000437, and hsa_0000280), because each subgroup independently regulated extra predicted autophagy-related genes. The total autophagy-related genes of each circRNA are listed in Supplemental Table 5, among which hsa_circ_0001747 appeared to be correlated with significantly more autophagy-related genes than the other circRNAs. Therefore, we ultimately selected hsa_circ_0001747 as our target gene for the next experimental verification.

### Knockdown of hsa_circ_0001747 promotes PCa proliferation in vitro and in vivo

**Fig. 7:**
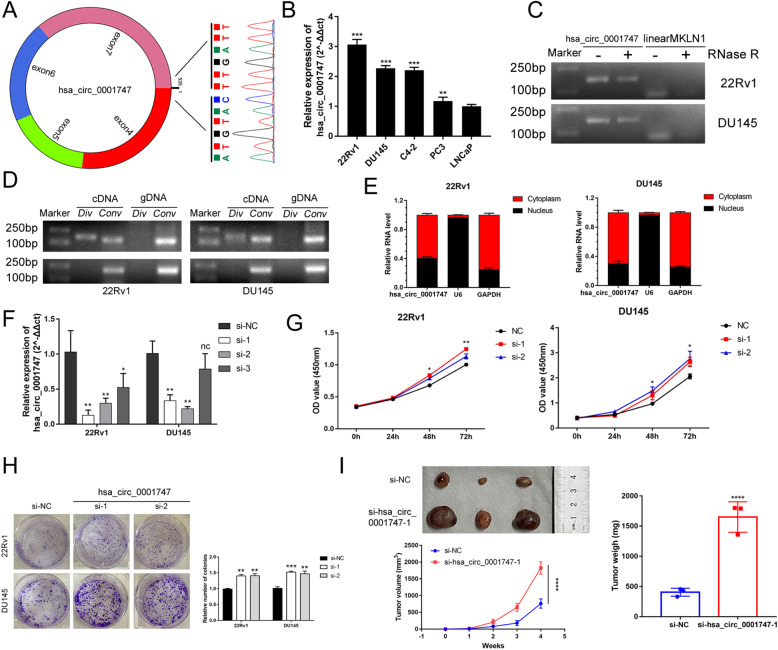
Characteristics of hsa_circ_0001747 and its functional effect on PCa. **A** Sanger sequencing identified the back-splicing site of hsa_circ_0001747. **B** Basal expression level of hsa_circ_0001747 in PCa cell lines. **C** Hsa_circ_0001747 was resistant to RNase R digestion. **D** Electrophoresis confirmed hsa_circ_0001747 was amplified in cDNA instead of gDNA samples. **E** Hsa_circ_0001747 was mainly distributed in the cytoplasm in PCa cells. **F** RT-qPCR verified the transfection efficiency of siRNAs targeting to hsa_circ_0001747 in DU145 and 22Rv1. **G** CCK-8 assay verified knockdown of hsa_circ_0001747 promoted PCa cells viability. **H** Knockdown of hsa_circ_0001747 facilitated colony formation in PCa cells. **I** Knockdown of hsa_circ_0001747 promoted the subcutaneous tumor growth in nude mice. Error bar indicates mean ± SD. *P < 0.05, **P < 0.01, ***P < 0.001, ****P < 0.0001.

We first input the chromosome position information of hsa_circ_0001747 to the circPrimer software (www.bioinf.com.cn) and found that hsa_circ_0001747 was transcribed from its corresponding host gene *MKLN1*, and its back-spliced junction was joined by exon 4 and exon 7, which was confirmed by Sanger sequencing (Fig. 7A). Then, we examined the basal expression of hsa_circ_0001747 in PCa cell lines, determining that hsa_circ_0001747 was highly expressed in 22Rv1 and DU145 cells (Fig. 7B). Using quantitative reverse transcription PCR (RT-qPCR) and electrophoresis, we verified that hsa_circ_0001747 could be amplified in complementary DNA (cDNA) instead of genomic DNA (Fig. 7C). Owing to its ring-shaped structure, hsa_circ_0001747 showed resistance to RNase R digestion (Fig. 7D). In addition, we detected the sub-location of hsa_circ_0001747 in DU145 and 22Rv1 cells, finding that it was pivotally located in the cytoplasm but only partially in the nucleus (Fig. 7E). To assess loss of function of hsa_circ_0001747 in PCa cells, we first designed three small interfering RNAs (siRNAs) targeting its back-spicing site and examined the transfection efficiency in 22Rv1 and DU145 cells. The results of RT-qPCR showed that both siRNA-1 and siRNA-2 significantly knocked down the expression of hsa_circ_0001747 in PCa cells (Fig. 7F). Cell Counting Kit-8 (CCK-8) assay and plate colony formation assay revealed that downregulation of hsa_circ_0001747 improved PCa cell viability and cell colony formation ability (Fig. 7G, H). Furthermore, we confirmed that knockdown of hsa_circ_0001747 facilitated subcutaneous tumor growth in male nude mice (Fig. 7I).

### Inhibition of hsa_circ_0001747 enhances PCa cell viability by augmenting autophagy

**Fig. 8:**
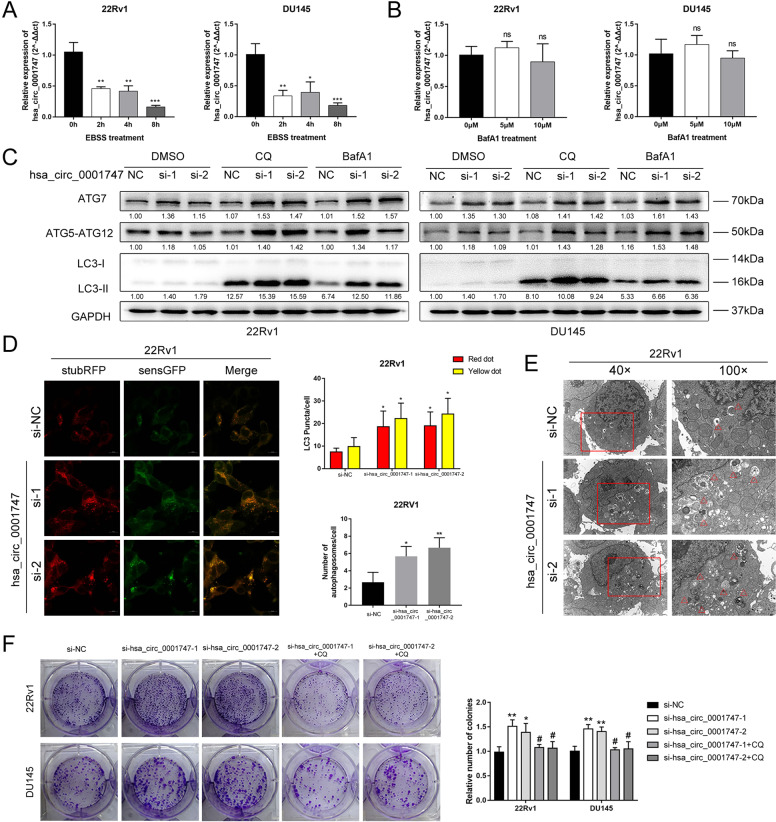
Downregulation of hsa_circ_0001747 promoted autophagy induced proliferation in PCa. **A** Hsa_circ_0001747 was downregulated in EBSS treatment. **B** Impediment of autophagy displayed no effect on the expression of hsa_circ_0001747. **C** Western blotting confirmed knockdown of hsa_circ_0001747 enhanced autophagy-related protein expression. **D** Knockdown of hsa_circ_0001747 facilitated the autophagy flux, including the autophagosomes (yellow dots) and autophagolysosomes (red dots) formation in 22Rv1 cells. **E** Knockdown of hsa_circ_0001747 promoted the formation of autophagosomes in 22Rv1 cells. **F** CQ treatment rescued the promotion of proliferation induced by knockdown of hsa_circ_0001747 in PCa. Error bar indicates mean ± SD. “ns” and “#” indicates not significant. *P < 0.05, **P < 0.01, ***P < 0.001.

Considering that hsa_circ_0001747 was an autophagy-related circRNA, as described above, we carried out autophagy induction and impairment experiments. Notably, hsa_circ_0001747 was markedly decreased in PCa cells treated with Earle’s balanced salt solution (EBSS) in a time gradient (Fig. 8A). However, when adding concentration gradient Bafilomycin A1 (BafA1) to block autophagy flux, hsa_circ_0001747 remained stable in both 22Rv1 and DU145 cells (Fig. 8B). This indicated that hsa_circ_0001747 may act as an upstream regulator in autophagy processing in PCa. *ATG5-ATG12* takes part in the initiation of autophagy and *ATG7* is responsible for the lipidation of *LC3*, which is vital to the biogenesis and maturation of autophagosome. Autophagy is a dynamic process that *LC3-II* will be degraded in a short time while initiating the autophagy, leading to the difficulty in the detection of the expression of *LC3* in western blotting assays [25]. Therefore, two specific vacuolar H^+^-ATPase inhibitors, i.e., chloroquine (CQ) and BafA1, which can inhibit the binding of autophagosomes and lysosomes to prevent *LC3-II* from degradation, were used for our further experiments [25]. In western blotting assays, we observed that when hsa_circ_0001747 was knocked down, the autophagy-related markers including *ATG5-ATG12*, *ATG7*, and *LC3-II* were modestly upregulated; however, such an association appeared to be significant with treatment of CQ and BafA1 (Fig. 8C). As described before that autophagy is dynamic, the result of western blotting only represented a transient state of autophagy in PCa cells; thus, we used the *LC3*-sensGFP-stubRFP assay to inspect the completed autophagy flux. SensGFP and stubRFP are both fluorescence-emitting markers in autophagosomes. In autolysosomes, the sensGFP signal is lost due to the low pH environment, only allowing for stubRFP to fluoresce. During the assay, we observed that autophagosomes (yellow dots) and autophagolysosomes (red dots) were accumulated when knocking down hsa_circ_0001747 (Fig. 8D and Supplemental Fig. S5A). In addition, the morphology of autophagosomes was identified in transmission electron microscopy, the number of which was consistent with that in western blotting and autophagy flux observation (Fig. 8E and Supplemental Fig. S5B). Importantly, the use of CQ to block autophagy could remarkably rescue promotion of cell proliferation of PCa cells induced by the inhibition of hsa_circ_0001747 (Fig. 8F).

## Discussion

Currently, the most threatening cancer among men is still PCa [26]. Among the various types of cancer, PCa is relatively indolent and generally progresses slowly in the early stages. BCR, which is characterized as consecutive PSA ≥ 0.2 ng/mL after a surgical procedure, is widely used to monitor the prognosis of patients with PCa [2]. However, if BCR occurs, PCa often becomes malignant or even metastatic to threaten lives, especially in cases with a high GS [27, 28]. Numerous studies have confirmed that autophagy might trigger this inevitable transformation; based on this, many researchers have identified various new autophagy regulatory networks in diverse types of cancer [29]. Fan et al. [30] revealed that autophagy can activate the *Wnt/β-catenin* signaling pathway and upregulate *MCT1* expression, thereby promoting metastasis and glycolysis in hepatocellular carcinoma. On the contrary, hypoxia and energy shortage commonly occur in hepatocellular carcinoma. Huang et al. [31] found that starvation induces *mTORC2-AKT* signaling pathway-enhanced *OXCT1*-mediated ketone body catabolism to raise cellular ATP levels and inhibits autophagy-dependent cell death. Thus, to identify the modulating role of autophagy in PCa, we carried out an IHC assay to verify the correlation of the classic autophagy marker *MAP1LC3B* with PCa progression. The results of TMA revealed that the expression level of *MAP1LC3B* protein in PCa tissues was higher than that in non-cancerous prostate tissues. In addition, high expression levels of *MAP1LC3B* protein were correlated with high GS in PCa tissues, which implied that autophagy might promote PCa progression in patients who experienced BCR.

As described previously, the regulatory role of autophagy in cancers is far from understood. Thus, we targeted circRNA in our investigation, which has been reported to play a vital role in the oncogenesis, progression, and chemoresistance of cancers [29, 32]. Specifically, circRNAs are closely correlated with autophagy in the regulation of cancers [21]. It has recently been reported that hypoxia-induced circCCDC66 acts as a stimulator in the tumorigenesis of colorectal cancer via the miR-3140/autophagy pathway [33]. Moreover, upregulation of autophagy activated by circ_0006528 exhibited a protective effect against breast cancers and showed resistance to paclitaxel [34].

Hence, we comprehensively analyzed autophagy-related circRNAs using bioinformatics and statistical methods, and constructed a prognostic model to accurately predict the risk of BCR and its corresponding outcomes in patients with PCa. First, by using Pearson’s correlation analysis, we screened 160 autophagy-related circRNAs based on the expression of 210 autophagy-related genes and 347 circRNAs. Then we applied the tenfold cross-validation LASSO regression to construct the prognostic signature in 144 PCa samples and identified five autophagy-related circRNAs: hsa_circ_0000280, hsa_circ_0000437, hsa_circ_0001085, hsa_circ_0002100, and hsa_circ_0001747. The 144 patients with PCa were stratified into high-risk and low-risk groups based on a median risk score of −0.14, in which those with a high risk have shorter BCR-free time than those with low risk. Multivariate Cox regression analysis indicated that risk score was an independent risk factor in prognostic prediction. Consistently, ROC analysis demonstrated that our prognostic model was more reliable (AUC = 0.827) than existing prognostic factors, such as GS, pathological stage, and pre-operation PSA. In addition, the nomogram containing different clinicopathological factors (age, GS, T-stage, PSA) and the risk score were used to accurately estimate the prognosis of patients with PCa. Notably, the calibration plots showed similarity between the predicted 3- and 5-year BCR-free survival and the actual corresponding survival rates in the nomogram.

To select one signature for further experimental validation, we used KM plots, and univariate and multivariate Cox regression analysis among all five autophagy-related circRNAs, finding that hsa_circ_0001747 was the most relevant prognostic signature. After verifying its biological characteristics, we conducted a loss-of-function assay, western blotting, and autophagy flux observation; the results demonstrated that hsa_circ_0001747 was a key blocker in autophagy, thereby suppressing PCa growth, which was consistent with the current evidence [35–37]. It has been reported that autophagy is generally enhanced and regulated by different types of ncRNA, which is attributed to its promotion of PCa progression or therapeutic resistance. In most cases, circRNA functions as an miRNA sponge to regulate different kinds of downstream target genes [19, 33, 34], or it acts as a scaffold to interact with modulating proteins [18, 22]. An increasing number of studies have revealed that circRNA governs the coding potential to regulate tumorigenesis and progression in cancers [38, 39]. In our study, hsa_circ_0001747 possessed numerous miRNA binding sites, but it correlated negatively with major autophagy-related genes and inhibited the autophagy process. Thus, we hypothesized that hsa_circ_0001747 might not function as a competing RNA to regulate autophagy. We then explored the possible characteristics of hsa_circ_0001747 in the CircInteractome, CircBank, and CircRNADb databases. We found that hsa_circ_0001747 not only carries many predicted binding sites of different proteins, such as *HUR* and *FUS*—upstream regulators of autophagy [11, 40]—but also it has the potential to encode peptides (coding probability: 0.9038; hexamer score: 0.0813; Fickett score: 0.8560). However, according to the standards of CircRNADb, the likelihood of hsa_circ_0001747 encoding proteins was relatively low, because its R score is <1.6. Collectively, we assumed that hsa_circ_0001747 might regulate PCa autophagy by interacting with potential binding proteins. These results warrant future investigation of its role in PCa autophagy.

The current study has some limitations though. Our prognostic model was constructed based on a cohort of 144 patients with PCa; therefore, further validation is needed in a larger external clinical sample. We will regularly track updates in related circRNA datasets for PCa, to improve the reliability of our current study findings. In addition, further in vitro and in vivo experiments will be needed to explore the specific mechanism of hsa_circ_0001747 in regulating autophagy in PCa.

Despite the above limitations, we believe that our prognostic model based on autophagy-related circRNA signatures will be a valuable supplement to the current evaluation system for the prognosis of patients with PCa. Furthermore, we have experimentally verified that autophagy level is positively related to the GS in PCa and we confirmed that autophagy enhancement by knockdown of hsa_circ_0001747 boosts the aggressiveness of PCa. The sum of these results suggested that using the prognostic model to identify subtypes of patients with PCa may be a promising therapeutic alternative for those with high risk scores.

In summary, our study showed that the autophagy-related circRNA signature could accurately predict BCR-free survival in patients with PCa with high and low risk. In addition, the constructed nomogram, constructed by integrating five autophagy-related circRNA signatures with other clinicopathological features, provided accurate and personalized BCR-free survival prediction. Finally, we selected hsa_circ_0001747 for further experimental validation and found that it was downregulated in PCa and potentially inhibited PCa proliferation by suppressing autophagy activation.

## Materials and methods

### Data processing

The circRNA and mRNA transcriptome (in FPKM) of 144 patients with PCa and clinical information was downloaded from GSE113124 in the Gene Expression Omnibus (GEO) database (http://www.ncbi.nlm.nih.gov/geo/). The differential circRNAs in 25 pairs of PCa and adjacent normal tissue samples were obtained from Vo et al. [41]. We used the normalizeBetweenArrays function of the “Limma” package to normalize circRNA expression profiles in GSE113124 and circRNAs with missing data that were excluded. R software version: 4.0.2 was used for all analyses in the study.

### Identification of autophagy-associated circRNAs

We downloaded 232 autophagy-related genes from HADb (http://www.autophagy.lu/index.html). We removed ten duplicated autophagy-related genes in the HADb and 12 undetected ones in GEO113124. The intersection (347 circRNAs) of 1199 circRNAs (FPKM > 0.5 in GSE113124) and 877 differentially expressed circRNAs [41] (absolute values of log2-fold change > 1 and FDR < 0.05) was identified. The 347 overlapped circRNAs’ ID was transformed into circBase (http://www.circbase.org/) ones based on the chromosomal location (Supplemental Table S1). Pearson’s correlation coefficients were used to explore the correlation between circRNAs and the filtrated 210 autophagy-related genes. Autophagy-related circRNAs were determined according to the criteria that the absolute value of the correlation coefficient was >0.3 (|*r*| > 0.3) and the *P*-value < 0.05.

### Construction and assessment of prognostic signature

LASSO regression is a machine learning algorithm for regression based on linear relationship assumption with a regularization L1 penalty. LASSO regression with *k*-fold cross-validation has been widely used as a dimension reduction strategy, in which the sample is randomly divided into *k* equal-sized subsamples with one subsample as validation data and the remaining *k* − 1 subsamples as training data. We used tenfold cross-validation LASSO regression in the “glmnet” package (nlambda parameter = 100) to lower the number of autophagy-associated circRNAs and construct the BCR prognostic signature in 144 patients with PCa. We calculated the risk score of each patient using the following formula:

$${\mathrm{Risk}}\;{\mathrm{score}} = \mathop {\sum}\nolimits_{i = 1}^N {{\mathrm{Coef}}_{i} \ast \left( {\mathrm{Expression}}\;{\mathrm{level}}\;{\mathrm{of}}\;{\mathrm{circRNA}}_{\rm{i}} \right)}$$

*N* is equal to the number of enrolled circRNAs and Coef_*i*_ represents the LASSO regression coefficients. The 144 patients with PCa from GSE113124 were divided into high- and low-risk groups, according to the median risk score. We used KM survival curves in the “survminer” package to compare the BCR of high- and low-risk patients, and the AUC using the “pROC” package to assess the predictive capacity of the signature and clinicopathological characteristics. Univariate and multivariate Cox analysis and KM plots were used to analyze the correlation between five autophagy-related circRNAs and BCR status. We constructed a nomogram and calibration plots based on the prognostic signature and clinical data using the “rms” R package. The c-index and calibration curves (1000 bootstrap samples) reflected the discriminative ability and accuracy of the nomogram.

### Tissue microarray and immunohistochemistry

TMA (*n* = 96, including 80 primary PCa tissues and 16 non-cancerous prostate tissues) was used in this study (PR1921a; Alenabio Biotech, Xi’an, China). All samples were anonymously handled according to ethical and legal standards. IHC staining was carried out using an UltraSensitive™ SP (mouse/rabbit) IHC kit (catalog number KIT-0305; MX Biotechnologies, Fuzhou, China). Briefly, The TMA slices were deparaffinized with dimethylbenzene and rehydrated in a gradient decent ethanol solution; after antigen retrieval with citrate buffer and inhibition of endogenous peroxidase with H_2_O_2_, the slices were incubated with primary antibody, followed by incubation with *Streptomyces* polyperoxidase-conjugated secondary antibody and nucleus staining with Diaminobenzidine. Finally, the slices were dehydrated, transparent, and encapsulated by neutral resin. The consequence of staining was evaluated by the intensity and percentage of stained cells by two pathologist independently. The intensity of staining was 0 (none), 1 (weak), 2 (moderate), and 3 (strong); the positive staining percentage was scored as 1 (0–10%), 2 (11–50%), 3 (51–75%), and 4 (75–100%). Final IRS scores were calculated by adding the percentage of positive cells to the intensity score. The detailed procedure can be referred to our previous study [42].

### Cell culture and treatment

All PCa cell lines, including 22Rv1, DU145, C4–2, PC-3, and LNCaP, were obtained from the National Collection of Authenticated Cell Cultures. DU145 and PC-3 were cultured in Dulbecco’s modified Eagle’s medium, and 22Rv1, C4–2, and LNCaP were cultivated in RPMI-1640 medium. Culture media contained 10% fetal bovine serum and 1% double antibiotics (Penicillin and Streptomycin). All cell lines were maintained at 37 °C and 5% CO_2_. For treatment with EBSS (24010043, GIBCO), 22Rv1 and DU145 were maintained in EBSS with or without 50 nM CQ (C6628-25G, 10 Sigma) or 10 μM BafA1 (B1793, Sigma) for 6 h.

### RNA extraction, RNase R treatment, and RT-qPCR

Total RNA was extracted from 22Rv1 and DU145 cells using Trizol reagent (15596018, Takara). Subsequently, total RNA was divided into two equal parts: one was treated with RNase R (R0301, Geneseed) for 37 °C to isolate the purified circRNAs and the other was used in the next step. Both of these two partial RNAs were then reverse-transcribed into cDNA using HiScript II Q RT SuperMix (R223-01, Vazyme). RT-qPCR was carried out using the SYBR Green Realtime PCR Master Mix (QPK-201, TOYOBO) in Stratagene Mx3000P (Aligent). glyceraldehyde 3-phosphate dehydrogenase (*GAPDH*) was used as an internal control. All experiments were carried out with three replicates. The primers of hsa_circ_0001747 and *GAPDH* are listed in Supplemental Table 2.

### Western blot and antibodies

All proteins were extracted from transfected cell lines using the RIPA lysis buffer and relative concentrations were quantified with a NanoDrop 2000 spectrophotometer. Briefly, the cell lysates were electrophoresed with 12% SDS-polyacrylamide gels (P0012AC, Beyotime) and the targeted bands were fully transferred onto polyvinylidene difluoride (IPVH00010, Merck Millipore) membranes; after blocked with 5% fat-free milk, the membranes were incubated with primary antibodies, followed by incubation with secondary antibodies and observation under chemiluminescence system. The detailed procedure can be referred to our previous study [42]. All experiments were carried out with three replicates. Primary antibodies used were as follows: *ATG5* (sc-133158, Santa Cruz), *ATG7* (sc-376212, Santa Cruz), *MAP1LC3B* (*LC3B*, NB100–2220, Novus), and *GAPDH* (60004-1-Ig, Proteintech). The intensity of each indicated protein band was quantified by measuring its grayscale via using the Image J software (version 1.8.0, NIH, USA). The targeted protein bands were first normalized to the reference (*GAPDH*) and then compared with the first band in the same row, respectively. Finally, statistical analysis was performed in each group (dimethyl sulfoxide, CQ, and BafA1), respectively.

### Cell transfection, cell viability, and plate colony formation assay

We seeded 1 × 10^5^ cells in six-well plates for fusion of 50–60% for 24 h; then cells were transfected with siRNAs mixed with siRNA-Mate (GenePharm) for 72 h and transfection efficiency was verified via RT-qPCR. We seeded 2000 transfected cells/well in 96-well plates, and then used CCK-8 reagent (MA0218-5, Meilunbio) to detect the viability of both cell lines at 24, 48, and 72 h. For plate colony formation assays, 500 transfected cells/well were added to six-well plates followed by treatment with CQ or phosphate-buffered saline (PBS) over the following 14 days. The colonies were finally fixed and stained with 0.1% crystal violet. All experiments were carried out with three triplicates. siRNAs targeting the back-splicing junction of hsa_circ_0001747 are shown in Supplemental Table 2.

### Autophagosome verification and autophagy flux observation

For autophagosome verification, 1 × 10^6^ transfected cells were collected and cell precipitates were fixed with 2% glutaraldehyde at 4 °C for more than 15 min. After washing with PBS three times for 10 min each, samples were post-fixed with 1% OsO_4_ followed by an ascending gradient dehydration step of ethanol and infiltration with propylene oxide. After performing ultrathin sectioning and staining with 3% lead citrate-uranyl acetate, samples were observed under an electron microscope (HT-7800, Hitachi High-tech). For autophagy flux detection, *LC3*-sensGFP-stubRFP-transfected PCa cells were fixed with 4% paraformaldehyde for more than 20 min after autophagy induction for 8 h; then, autophagy flux was observed using confocal fluorescence microscopy (Leica, Germany). In merged images, yellow spots represent autophagosomes and red spots represent autolysosomes in individual images.

### Xenograft models

Four-week-old male C57BL/6 nude mice were obtained from Guangdong Experimental Animal Center (Guangzhou, China). Six male C57BL/6 mice were randomized into two groups and then subcutaneously injected with 5 × 10^6^ DU145 cells in the hindquarters. The growth of implanted PCa tumors was monitored by measuring volumes each week. Finally, the mice were killed and xenografts were measured and photographed.

### Statistical analyses

R software version 4.0.2 (The R Project for Statistical Computing, Vienna, Austria) was used for all bioinformatics statistical analysis. Pearson’s correlation coefficients were used to analyze correlations between circRNAs and autophagy-related RNA. KM plots and Cox analysis were performed using the “survival” and “survminer” packages, and forest plots were performed with “forestplot” package. GraphPad Prism 7.0 (GraphPad, La Jolla, CA, USA) was used to analyze the statistical results of RT-qPCR, cell functional assays, and animal experiments. Comparisons for two groups or multiple groups were performed using Student’s *t*-test or one-way analysis of variance. All results were displayed as mean ± SD; all tests were two-sided and the *P*-value < 0.05 was considered statistically significant.

## Supplementary information

Supplemental Figure Legends

Supplemental Table 1

Supplemental Table 2-4 & Figure 1-6

Supplemental Table 5

## Data Availability

The datasets generated and/or analyzed during the current study are available in GEO database https://www.ncbi.nlm.nih.gov/geo/query/acc.cgi?acc=GSE113124.
