# Correction: Autophagy-related circRNA evaluation reveals hsa_circ_0001747 as a potential favorable prognostic factor for biochemical recurrence in patients with prostate cancer

**DOI:** 10.1038/s41419-021-04051-6

**Published:** 2021-08-04

**Authors:** Chuanfan Zhong, Kaihui Wu, Shuo Wang, Zining Long, Taowei Yang, Weibo Zhong, Xiao Tan, Zixian Wang, Chuanyin Li, Jianming Lu, Xiangming Mao

**Affiliations:** 1 Department of Urology, Zhujiang Hospital, Southern Medical University, Guangzhou, China; 2 Irvine Valley College, Irvine, CA USA

**Keywords:** Tumour biomarkers, Macroautophagy

Correction to: *Cell Death and Disease* 10.1038/s41419-021-04015-w, published online 22 July 2021

The original version of this article unfortunately contained a mistake. Due to a typesetting error Chuanyin Li and Jianming Lu were omitted as the co-corresponding authors. In addition, their names were also omitted in the section “Correspondence and requests for materials should be addressed to”. The correct section should read “Correspondence and requests for materials should be addressed to C.L., J.L. or X.M.”. We apologize for these errors. The original article has been corrected.

