# Supplementary material for: Autophagy-related circRNA evaluation reveals hsa_circ_0001747 as a potential favorable prognostic factor for biochemical recurrence in patients with prostate cancer
Source: Cell Death Dis. 2021 Jul 22;12(8):726. doi: 10.1038/s41419-021-04015-w (PMC8298711; doi:10.1038/s41419-021-04015-w)
Supplement: Supplementary file 1 — Supplemental Figure Legends [file 41419_2021_4015_MOESM1_ESM.docx]

**Supplemental Figure 1** LASSO regression analysis of autophagy-related circRNAs. (A) Differential circRNAs in 25 pairs of PCa and adjacent normal tissues. (B) LASSO coefficient profiles of autophagy-related circRNAs. (C) 10-fold cross-validation results that identified optimal values of the penalty parameter λ.

**Supplemental Figure 2** Relative expression of five autophagy-related circRNAs in PCa and adjacent normal tissues. (A) Hsa_circ_0001747, (B) hsa_circ_0000437, (C) hsa_circ_0000280, (D) hsa_circ_0002100, and (E) hsa_circ_0001085.

**Supplemental Figure 3** KM plot analysis of five autophagy-related circRNAs in patients with PCa. (A) Hsa_circ_0001747 (with statistical significance) and (D) hsa_circ_0002100 (without statistical significance) display favorable in BCR-free survival in patients with PCa. (B) Hsa_circ_0000437, (C) hsa_circ_0000280 (with statistical significance) and (E) hsa_circ_0001085 (without statistical significance) display favorable in BCR-free survival in patients with PCa.

**Supplemental Figure 4** Univariate (A) and multivariate (B) Cox regression analysis of the autophagy-related circRNAs.

**Supplemental Figure 5** Autophagosomes morphology identification and autophagy flux detection in confocal microscopy. (A) Knockdown of hsa_circ_0001747 facilitated the autophagy flux, including the autophagosomes (yellow dots) and autophagolysosomes (red dots) formation in DU145 cells. (B) Knockdown of hsa_circ_0001747 promoted the formation of autophagosomes in DU145 cells. Error bar indicates mean ± SD. *P<0.05.

**Supplemental Figure 6** Biological prediction of potential interactive proteins and possibility of encoding protein of hsa_circ_0001747. (A) The potential interactive proteins of hsa_circ_0001747 in CircInteractome Database. (B) The possibility of encoding protein of hsa_circ_0001747 was predicted in CircRNADb Website.
