# Supplementary material for: Autophagy-related circRNA evaluation reveals hsa_circ_0001747 as a potential favorable prognostic factor for biochemical recurrence in patients with prostate cancer
Source: Cell Death Dis. 2021 Jul 22;12(8):726. doi: 10.1038/s41419-021-04015-w (PMC8298711; doi:10.1038/s41419-021-04015-w)
Supplement: Supplementary file 3 — Supplemental Table 2-4 & Figure 1-6 [file 41419_2021_4015_MOESM3_ESM.docx]

| Supplemental Table 2 RT-qPCR primers and siRNAs used in the study | | |
| --- | --- | --- |
|  | Forward (5'-3') | Reverse (5'-3') |
| hsa_circ_0001747 | AAACACTTCAGACAACACAACTA | GAATCAAGTACATTTACAGCCTT |
| GAPDH | TGCCATGTAGACCCCTTGAA | GTCCACCACCCTGTTGCT |
|  |  |  |
|  | Sense (5'-3') | Anti-sense (5'-3') |
| siRNA control | UUCUCCGACGUGUCACGUTT | AGGUGACACGUUCGGAGAATT |
| si-hsa_circ_0001747-1 | GCUGUAAAUGUACUUGAUUCUTT | AGAAUCAAGUACAUUUACAGCTT |
| si-hsa_circ_0001747-2 | GAAAGGCUGUAAAUGUACUUTT | GAAAAGGCUGUAAAUGUACUUTT |

Supplemental Table 3 Univariate and multivariate Cox regression analysis of prognostic model and clinicopathological characteristics (Cox proportional hazards regression model）

| Viable | Univariate analysis | | | | Multivariate analysis | | |
| --- | --- | --- | --- | --- | --- | --- | --- |
|  | HR | 95% CI | P value | HR | | 95% CI | P value |
| Age | 1 | 0.96-1.1 | 0.55 |  | |  |  |
| PSA | 1 | 0.94-1.1 | 0.65 |  | |  |  |
| Gleason | 3.2 | 1.8-5.5 | 3.3e-05 | 3.3 | | 1.8-6.1 | 0.00019 |
| Risk score (High vs. Low) | 11 | 3.3-36 | 9.3e-05 | 10 | | 3.1-34 | 0.00013 |
| T stage | 1.4 | 0.92-2 | 0.12 |  | |  |  |

Abbreviations: HR: hazard ratio; CI: confidence interval.

Supplemental Table 4 Univariate and multivariate Cox regression analysis of five autophagy-related circRNAs (Cox proportional hazards regression model）

| Viable | Univariate analysis | | | | Multivariate analysis | | |
| --- | --- | --- | --- | --- | --- | --- | --- |
|  | HR | 95% CI | P value | HR | | 95% CI | P value |
| hsa_circ_0000437 | 1.14 | 1.01-1.29 | 0.0282 |  | |  |  |
| hsa_circ_0000280 | 2.19 | 1.18-1.07 | 0.0131 | 2.20 | | 1.26-3.83 | 0.0055 |
| hsa_circ_0002100 | 0.41 | 0.21-0.79 | 0.0075 | 0.51 | | 0.26-1.00 | 0.0511 |
| hsa_circ_0001747 | 0.30 | 0.15-0.60 | 0.0007 | 0.22 | | 0.09-0.55 | 0.0011 |
| hsa_circ_0001085 | 2.40 | 1.39-4.15 | 0.0018 | 2.39 | | 1.39-4.11 | 0.0017 |

Abbreviations: HR: hazard ratio; CI: confidence interval.


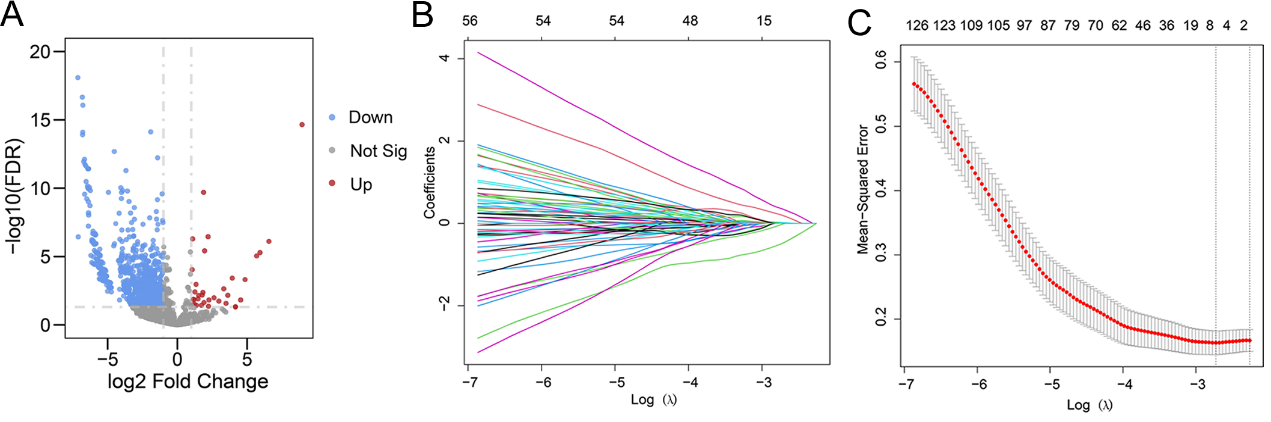


Supplemental Figure 1 LASSO regression analysis of autophagy-related circRNAs.


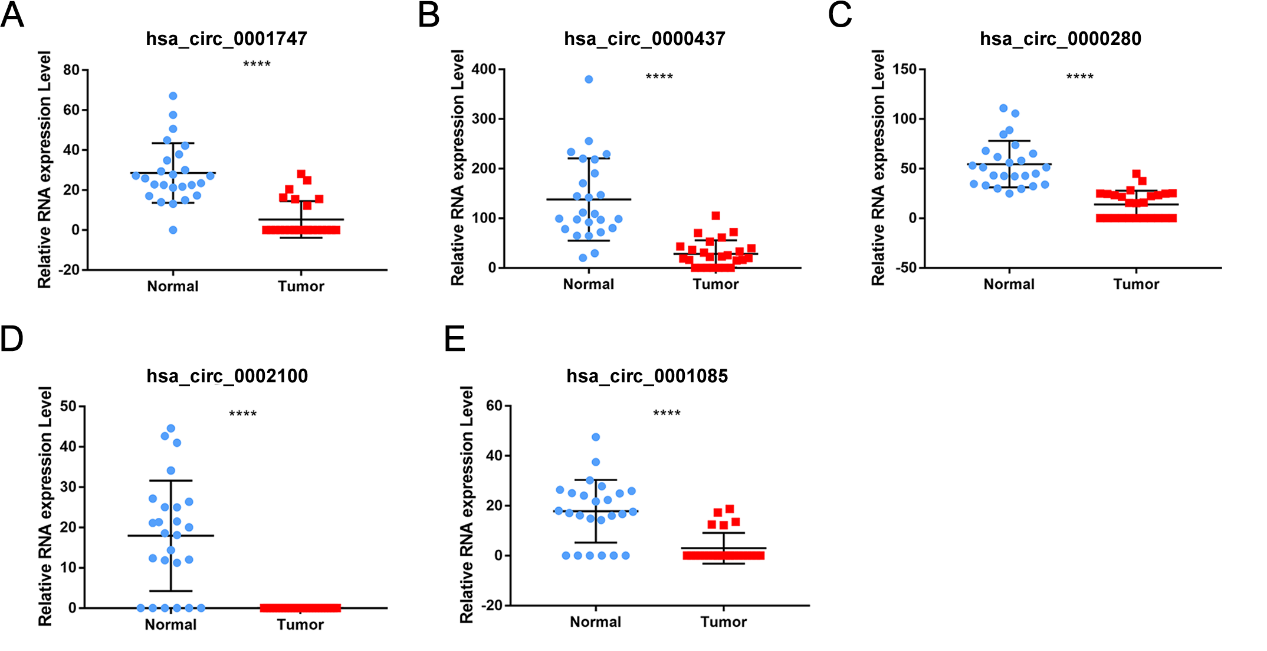


Supplemental Figure 2 Relative expression of five autophagy-related circRNAs in PCa and adjacent normal tissues.


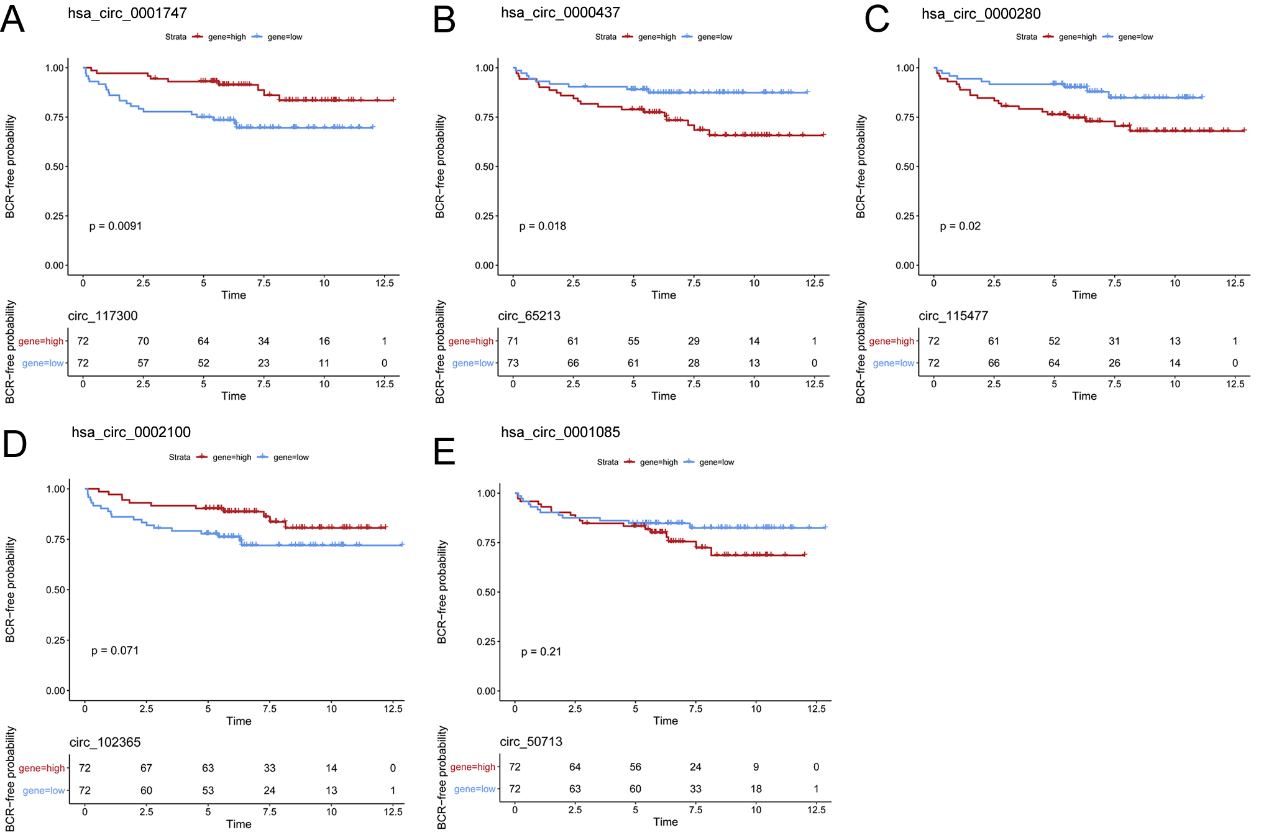


Supplemental Figure 3 KM plot analysis of five autophagy-related circRNAs in patients with PCa.


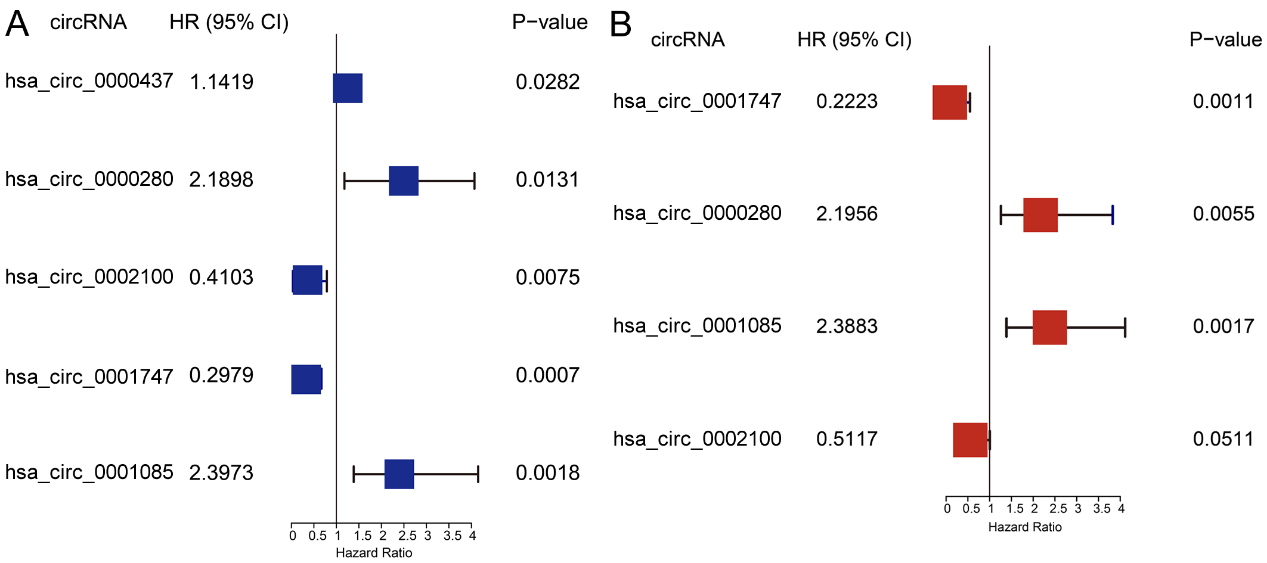


Supplemental Figure 4 Univariate and multivariate Cox regression analysis of the autophagy-related circRNAs.


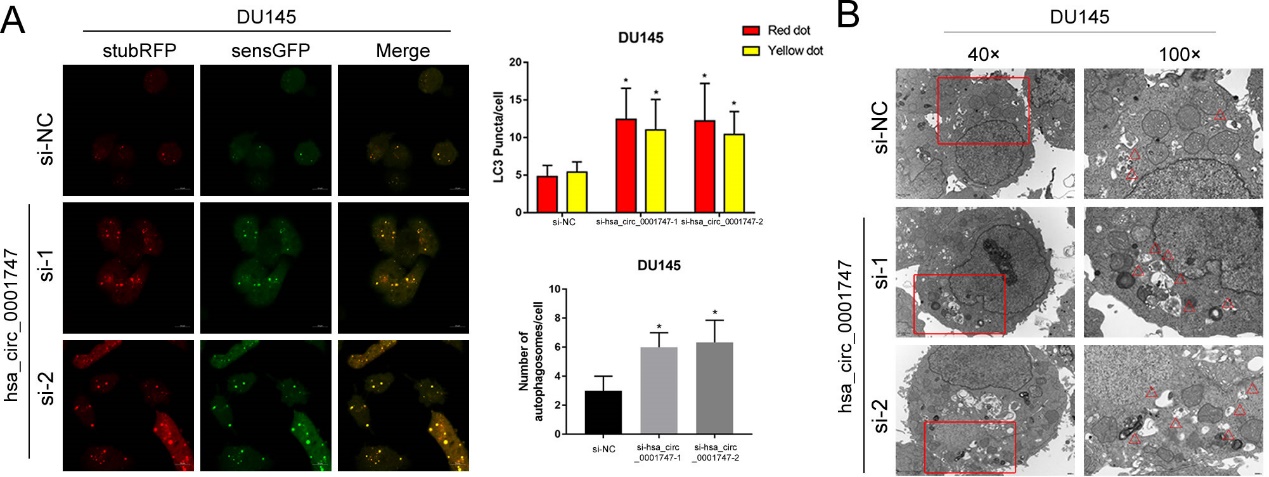


Supplemental Figure 5 Autophagosomes morphology identification and autophagy flux detection in confocal microscopy.


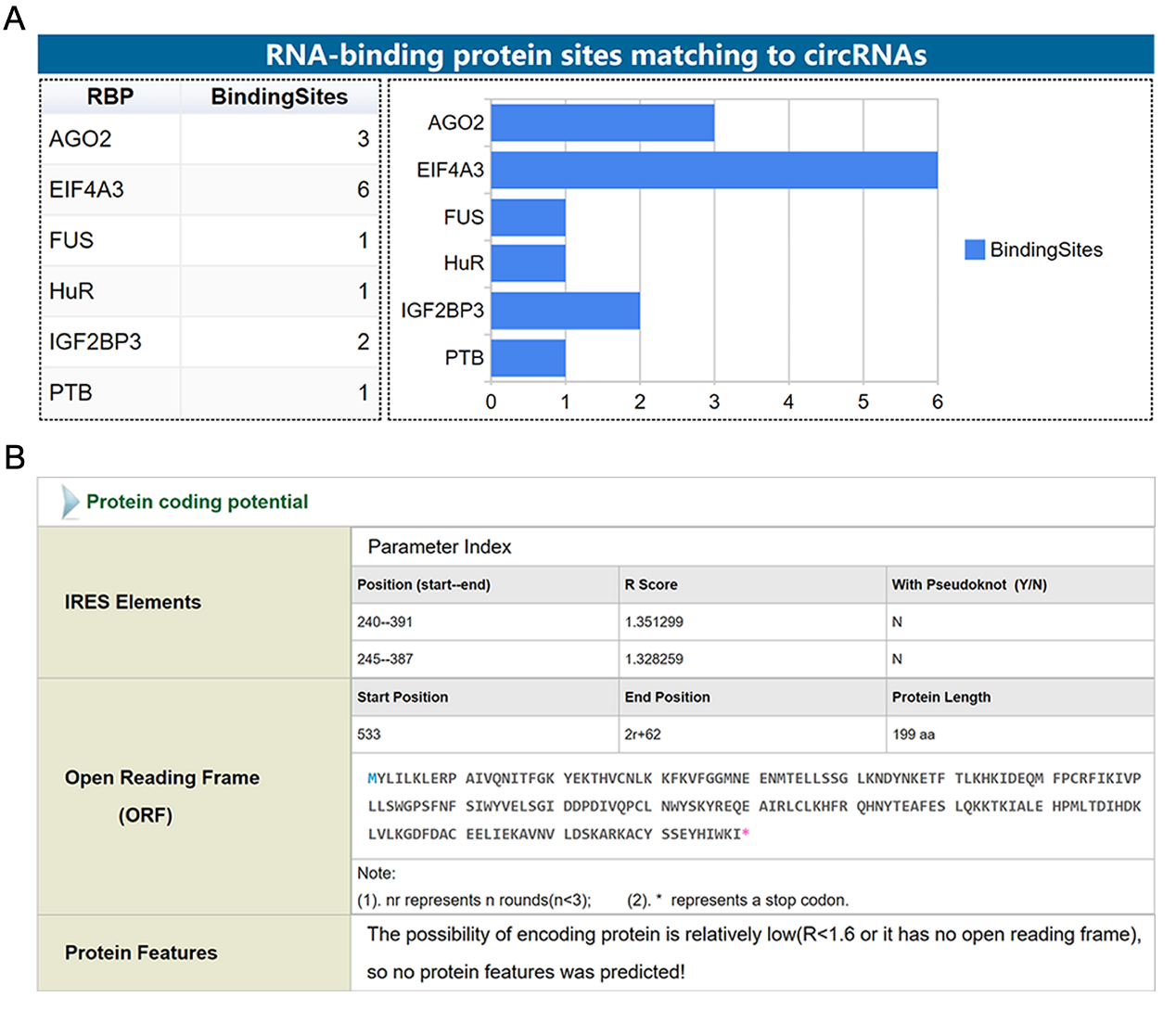


Supplemental Figure 6 Biological prediction of potential interactive proteins and possibility of encoding protein of hsa_circ_0001747.
